# Enhanced Production and Characterization of a Solvent Stable Amylase from Solvent Tolerant *Bacillus tequilensis* RG-01: Thermostable and Surfactant Resistant

**DOI:** 10.1155/2014/972763

**Published:** 2014-10-27

**Authors:** Soni Tiwari, Neha Shukla, Pooja Mishra, Rajeeva Gaur

**Affiliations:** Department of Microbiology, Centre of Excellence, Dr. Ram Manohar Lohia Avadh University, Faizabad, Uttar Pradesh 224001, India

## Abstract

Ten bacterial strains isolated from the soil samples in the presence of cyclohexane were screened for amylase production. Among them, culture RG-01 was adjudged as the best amylase producer and was identified as *Bacillus tequilensis* from MTCC, Chandigarh. The isolate showed maximum amylase production (8100 U/mL) in the presence of starch, peptone, and Ca^2+^ ions at 55°C pH 7.0 within 24 h of incubation. The enzyme was stable in the presence of n-dodecane, isooctane, n-decane, xylene, toluene, n-hexane, n-butanol, and cyclohexane, respectively. The presence of benzene, methanol, and ethanol marginally reduced the amylase stability, respectively. The enzyme was showed it 100% activity at 55°C and pH 7.0 with 119% and 127% stability at 55°C and pH 7.0, respectively. The enzyme was also stable in the presence of SDS, Tween-40, Tween-60, and Tween-80 (1%) and was found stimulatory effect, respectively. Only Triton-X-100 showed a moderate inhibitory effect (5%) on amylase activity. This isolate (*Bacillus tequilensis* RG-01) may be useful in several industrial applications owing to its thermotolerant and organic solvents and surfactants resistance characteristics.

## 1. Introduction

In modern times, the products of biological origin, particularly enzymes, are attracting the attention of researchers. Their role in several biological and commercial processes has been duly emphasized. Among all the enzymes, *α*-amylases constitute a class of industrial enzyme having approximately 30% of the world enzyme production [1] and represent one of the three largest groups of industrial enzymes and account for approximately 25% of total enzymes sales [2] and are an important enzyme, particularly in the process of starch or glycogen hydrolysis.

The amylases can be derived from several sources such as plants, animals, and microbes. The major advantage of using microorganisms for production of amylases is in economical bulk production capacity and microbes are also easy to manipulate to obtain enzymes of desired characteristics [3, 4]. Based on their mode of action, they are further classified into three categories: *α*-amylases, *β*-amylases, and glucoamylases. All amylases are glycoside hydrolyser and act on *α*-1,4 glycosidic bonds [5]. Industrially, *α*-amylase is used particularly in starch liquefaction, brewing, textile, pharmaceuticals, paper, detergents, drugs, toxic wastes removal, and oil drilling [6]. Since *α*-amylases are active over a broad pH (5–9) and temperature (35–105°C) ranges, they are worldwide center of attraction for researchers. Many microorganisms like fungi, yeast, bacteria, and actinomycetes produce this enzyme; however, enzyme from fungal and bacterial sources has dominated applications in industrial sectors [7]. However, bacterial amylases are preferred as they grow rapidly, need less space, can be easily maintained, and are accessible for genetic manipulations. The important amylase producing bacteria are species of* Bacillus, Pseudomonas, Halomonas, Arthrobacter, *and* Serratia*. Among the bacterial sources,* Bacillus subtilis*,* B*.* stearothermophilus, B*.* amyloliquefaciens, B*.* licheniformis, B*.* acidocaldarius, Bifidobacterium *bifidum, and* B*.* macerans* [3, 8, 9] play an important role in production of amylase. Several species of* Bacillus* are industrially employed to produce thermostable amylase as they grow easily under extreme pH and temperature conditions.

The application of an amylase in industrial reactions depends on its unique characteristics, such as its action pattern, substrate specificity, major reaction products, optimal temperature, and optimal pH [10]. They are mainly employed for starch liquefaction to reduce their viscosity, production of maltose, oligosaccharide mixtures, high fructose syrup, and maltotetraose syrup. In detergents production, they are applied to improve cleaning effect and are also used for starch desizing in textile industry [11]. Generally, production of this enzyme has been carried out by submerged fermentation [8] because of the ease of sterilization and process control that is easier to engineer in these systems. The purpose of this study is to isolate a novel thermotolerant amylase producing bacteria in the presence of organic solvent. Simultaneous optimization and characterization of different parameters were also performed in this study.

## 2. Materials and Methods

### 2.1. Isolation, Screening, and Identification of Thermotolerant Amylase Producing Bacteria

The soil samples were collected aseptically from different sites of University campus to isolate amylase producing bacteria. One g soil was suspended in 9.0 mL sterile distilled water, agitated for a min and 0.1 mL suspension was spread over nutrient starch agar plates (pH 7.0) containing 2.0% starch; 0.5% peptone; 0.3% beef extract; 0.5% NaCl; 2% agar. The inoculated plates were overlaid with 7.0 mL of cyclohexane and incubated at 55°C till sufficient growth appeared. After sufficient growth incubated plates were overlaid with Gram's iodine reagent (0.01 M I_2_-KI solutions). If a strain was amylolytic, then it started hydrolyzing the starch present in the surrounding and in the zone degradation there was no blue color formation. Selection was done as per colonies with and without clear and transparent zone as amylase producing (Amy+) and amylase nonproducing (Amy−) strain, respectively. Bacterial colonies showing clear zones were selected, streaked twice on starch agar plates for purification, and maintained as pure culture over nutrient agar slants (pH 7, 4°C). The isolate having maximum clearance zone was selected for further studies. The selected bacterial isolate RG-01 was identified by morphological and biochemical characterization as per Bergey's Manual of Systematic Bacteriology [12]. The identity of RG-01 was authenticated from the Institute of Microbial Technology (IMTECH), Chandigarh, India, based on the phenotypic (16S rDNA) and biochemical tests.

### 2.2. Inoculum Preparation

Mother culture was prepared by inoculating one full loop of 24 h grown culture on starch agar plate in 50 mL starch broth and incubated at 55°C for 24 h to achieve active exponential phase containing 3.2 × 10^8^ cfu/mL. Suitable amount (0.5%, v/v) of this cell suspension was used to inoculate the test flasks.

### 2.3. Crude Enzyme Preparation

To obtain crude enzyme, 24 h old cultures were transferred to microcentrifuge tubes and centrifuged at 10000 rpm for 10 min. Cells were discarded and resultant supernatant was used as the crude enzyme for various enzyme assays (activity).

### 2.4. *α*-Amylase Assay (Activity)

The activity of *α*-amylase was assayed by measuring the reducing sugar released by reaction on starch [13, 14]. Amylase assay was done by using a reaction mixture consisting of 500 *μ*L of substrate solution (1.0% soluble starch in 1.0 M sodium phosphate buffer pH 7.0), 100 *μ*L of the enzyme solution, and 1 mL volume make up by adding 400 *μ*L distilled water. The reaction mixture was incubated for 10 min at 55°C. Reaction was stopped by adding 1 mL of alkaline copper tartrate solution. The reaction mixture was heated to 100°C for 10 min and cooled and then added to arsenomolybdate solution for color stabilization. Optical density of each sample with reaction mixture was taken at 620 nm in a spectrophotometer (Shimadzu, Japan). One unit of *α*-amylase activity was defined as the amount of enzyme that liberates 1.0 *μ*g of glucose (reducing end groups) per min/mL in 1.0 M sodium phosphate buffer (pH 7.0) with 1.0% (w/v) soluble starch as substrate at 55°C.

### 2.5. Culture Conditions and Medium Selection

The selected isolate RG-01 was grown in nutrient starch and modified (in our laboratory) nutrient starch broth. The modified nutrient starch broth contained 2.0% starch; 0.5% peptone; 0.3% beef extract; 0.5% NaCl; 0.05% MgSO_4_. To study the growth behavior and amylase production, 1.0 mL of mother culture having 0.8 OD (A_620_; 1 cm cuvette) containing 3.2 × 10^8^ cfu/mL was inoculated in 99 mL of broth (pH 7.0; adjusted with 1 N NaOH) in Erlenmeyer flasks and incubated at 55 ± 1°C on incubator shaker (100 rpm) for 24 h. At 4 h interval, bacterial growth was assessed by turbidity measurement at 620 nm. Each sample was centrifuged at 10,000 rpm (4°C) for 10 min and cell-free supernatant was assayed for amylase activity.

### 2.6. Optimization of Physicochemical and Nutritional Parameters for Amylase Production

The various process parameters influencing amylase production were optimized individually and independently of the others. Therefore, the optimized conditions were subsequently used in all the experiments in sequential order. For optimization, the basal medium was inoculated and incubated at different temperatures,* viz*, 35, 40, 45, 50, 55, 60, 65, 70, and 75°C under the standard assay conditions. The samples were withdrawn at every 4 h interval up to 48 h to study the effect of incubation periods. The influence of pH on the enzyme activity was determined by measuring the enzyme activity at varying pH values ranging from 5.0 to 12.0 at 55°C using different suitable buffers at concentration of 100 mM citrate buffer (pH 5.0-6.0, 1 M), phosphate buffer (6.0–7.5), Tris-HCl buffer (pH 8.0-9.0), and glycine-NaOH (9.5–11.0) under standard assay conditions. The growth medium was supplemented with different carbon sources,* viz*, starch (soluble), potato starch, wheat bran, rice bran, glucose, fructose, lactose, maltose, and sucrose (at the level of 2%, w/v). Different nitrogen sources (beef extract, yeast extract, peptone, urea, ammonium sulphate, ammonium chloride, and ammonium hydrogen phosphate 0.5% w/v) were also used for enzyme production. Thereafter, optimized carbon and nitrogen sources were further optimized at different concentrations.

### 2.7. Effect of Organic Solvents on Amylase Stability

Cell-free supernatant having maximum amylase activity was filtered with nitrocellulose membrane (pore size 0.22 *μ*m) and incubated with 30% (v/v) of different organic solvents,* viz*, n-dodecane, n-decane, isooctane, n-octane, xylene, n-hexane, n-butanol, cyclohexane, n-heptane, benzene, toluene, ethanol, methanol, and propanol for 7 days in screw crapped tubes at 55°C and 120 rpm. The residual amylase activity was estimated against the control, in which solvent was not present.

### 2.8. Effect of Metal Ions on Enzyme Activity and Stability

The effect of various metal ions (25 mM) on enzyme activity was investigated using FeSO_4_, CaCl_2_, KCl, NaCl, MgSO_4_, MnCl_2_, ZnSO_4_, CuSO_4_, HgCl_2_, and NiCl_2_. The enzyme was incubated with different metals at 55°C for 1 h to study metal ion stability and assayed under standard assay conditions. The effect of different concentrations (5 mM to 45 mM) of CaCl_2_, NaCl, and MgSO_4_ on amylase activity was also evaluated under standard assay conditions.

### 2.9. Effect of Surfactants on Enzyme Stability

The effect of various surfactants (1.0%) on enzyme activity was investigated using Triton-X-100, Tween-40, Tween-60, Tween-80, and SDS. The amylase sample was incubated with surfactants for 1 h at 55°C and then the residual activity (%) was tested under standard assay conditions.

### 2.10. SDS-PAGE Analysis

The enzyme supernatant with maximum enzyme activity along with marker was electrophoresed by sodium dodecyl sulphate-poly acrylamide gel electrophoresis in a 12.5% polyacrylamide gel according to the method of Laemmli [15]. Approximate molecular weight of the amylase was estimated by SDS-PAGE against the molecular mass markers, i.e., lysozyme (14.3 kDa), *β*-lactoglobulin (20 kDa), carbonic anhydrase (29 kDa), ovalbumin (43 kDa), bovine serum albumin (66 kDa), and phosphorylase B (97.4 kDa) (Sigma-Aldrich Pvt Ltd., USA) run with the samples.

### 2.11. Statistical Analysis

All experiments were carried out in triplicates and the results are presented as the mean of three independent observations. Standard deviation for each experimental result was calculated using Microsoft Excel.

## 3. Results and Discussion

### 3.1. Isolation, Screening, and Identification of Thermotolerant Organic Solvent-Resistant Amylase Producing Bacterial Cultures

Ten (10) bacterial isolates producing variable amylolytic zones on starch agar plates which stained with iodine solution were isolated from the soil samples in the presence of cyclohexane. The zones of clearance by isolates reflect their extent of amylolytic activity. Those having clearance zone greater than >1.5 cm were considered as significant. Among 10 bacterial isolates, 5 exhibited good amylase activity which was reassessed by loading their culture broth in the wells on starch agar plates which stained with iodine solution (pH 7.0). The culture broth of good amylase producers cleared more than >1.5 cm zone within 3-4 h of incubation at 55 ± 1°C, thereby indicating an extra-cellular nature of the amylase. The isolate RG-01, showing maximum clearance zone diameter, was selected for further studies.

**Figure 1:**
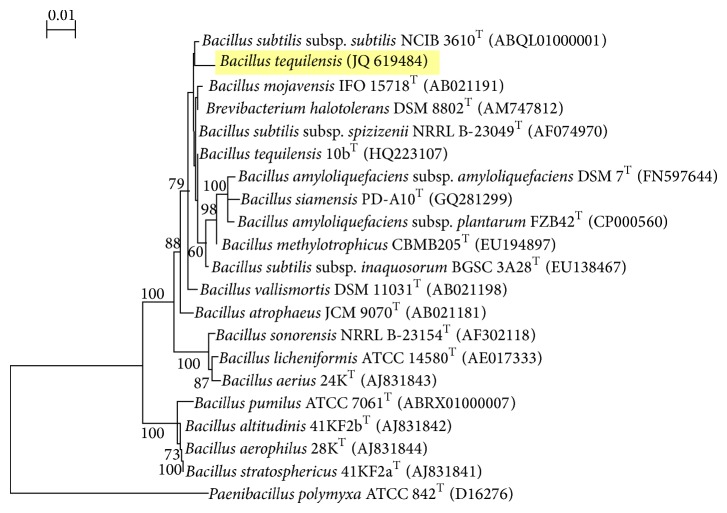
Phylogenetic tree showing relation between strain RG-01 and other* Bacillus* strains.

The efficient strain RG-01 was rod-shaped, Gram-positive, aerobe and facultative, motile, with positive Voges-Proskauer, catalase and oxidase test. It grew over a wide range of pH (5–12), temperature (15°–85°C), and NaCl concentration (0.0–16%), and it was able to hydrolyze gelatin, esculin, starch, Tween-20, and Tween-40 and produce acid from glucose. The strain was halotolerant as it grew in the presence of 0.0–12% NaCl but did not require salt for its physiological activities (Table 1). On account of morphological and biochemical characteristics, it was identified as* Bacillus *sp. by MTCC MTECH, Chandigarh, India. Analysis of 16S rDNA sequence (1444 bp) revealed its 99.3% homology with* Bacillus tequilensis *strains and was designated as* Bacillus tequilensis *RG-09. The 1444 bp 16S rDNA sequence was submitted to GenBank (JQ: 619484). The strain RG-09 was in the same cluster of phylogenetic tree (Figure 1) with different strains of* Bacillus tequilensis. *However, the 16S rDNA sequence analysis indicates that it is a different and novel strain of* Bacillus tequilensis*. The 16S rDNA is the most widely accepted gene employed for bacterial classification and identification. Gupta et al. [16] emphasized that the use of molecular markers like 16S rDNA as species-specific identification tool has been provided with a truly “microscopic” sensitivity down to single-cell detection.

### 3.2. Culture Conditions and Medium Selection

**Figure 2:**
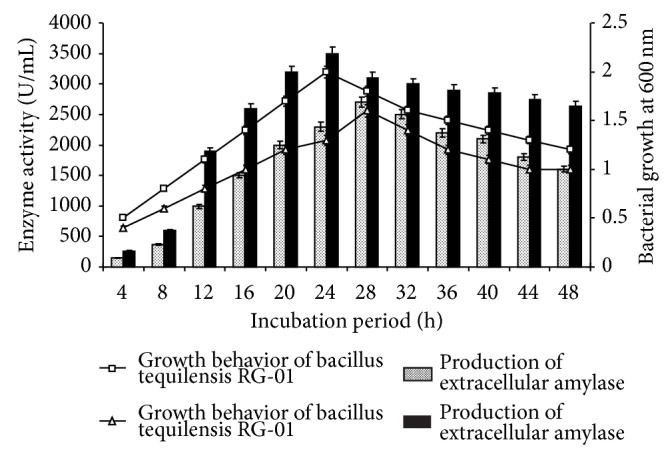
Growth behavior of* Bacillus tequilensis* RG-01 and production of extracellular amylase, respectively, in starch nutrient media and modified starch nutrient media at initial pH 7.0 and 55°C and 120 rpm during 48 h growth.

The RG-01 strain exhibited typical sigmoidal growth behavior in both culture media. In nutrient starch broth, stationary phase commenced at 24 h, while, in modified nutrient starch broth, the onset of stationary phase was at 20 h onward after a steep log phase. In both media, the stationary phase of bacterial growth witnessed maximum amylase production. However, the amylase production was the maximum (3500 U/mL) in modified nutrient starch medium at 20 h, while it was 2700 U/mL at 24 h in nutrient starch medium (Figure 2). Hence, modified nutrient starch medium was selected for further studies on amylase production.

### 3.3. Effect of Different Temperatures

**Figure 3:**
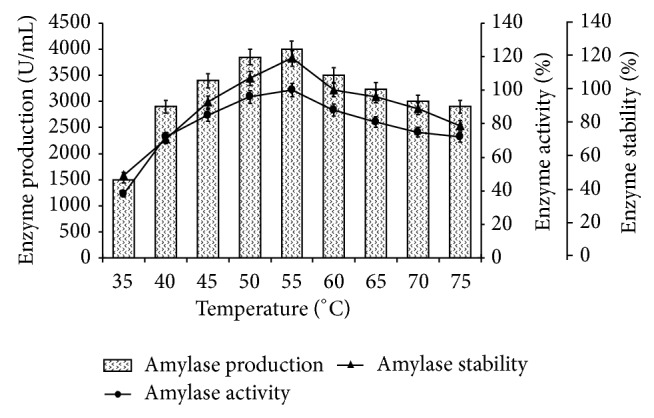
Effect of temperature on amylase production, activity, and stability. The flasks were inoculated with culture in the medium and were incubated at different temperature (35–75°C) for 24 h at pH 7.0. For enzyme activity, reaction mixture was incubated at different temperatures (35–75°C) and for stability enzyme was preincubated at respective temperatures for 1 h and reaction was conducted as standard assay method. Error bars presented mean values of ±standard deviation of triplicates of three independent experiments.

Effect of different temperatures,* viz*, 35°C to 75°C, was evaluated for amylase production, activity, and stability by* Bacillus tequilensis *RG-01 at different physicochemical and nutritional levels.* Bacillus tequilensis *RG-01 showed higher amylase production, activity, and stability (4000 U/mL, 100%, and 119%) at 55°C. This culture also sustains its activity and stability (81% and 96.3%) at 65°C. Further increase in temperature could not affect the amylase production by* Bacillus tequilensis *RG-01 (Figure 3). Similarly, Anto et al. [17] also reported that *α*-amylase production by* B. subtilis* at 70°C is 90% activity and was observed compared to the optimum enzyme activity at 55°C for liquefaction of starch. For *α*-amylase produced by a* Bacillus* isolate AS-1, 88, 85, and 44% of activity have been reported at 60, 70, and 80°C, respectively [18].

### 3.4. Effect of Different pH

**Figure 4:**
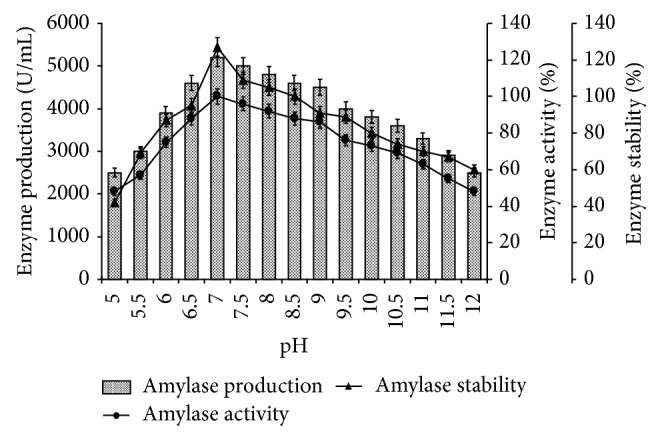
Effect of pH on amylase production, activity, and stability. The flasks were inoculated with culture and were incubated at different pH (5–12) for 24 h at 55°C. For enzyme activity, the reaction was assayed at respective pH and for stability enzyme was preincubated with buffers (100 mM, in ratio 1 : 1) of different pH (5–12) at 55°C for 1 h and assayed by standard assay method. Error bars presented mean values of ±standard deviation of triplicates of three independent experiments.

Among the physical parameters, the pH of the growth medium plays an important role by inducing morphological change in the organism and in enzyme secretion [19, 20]. Different pH,* viz*, 5.0 to 12.0 in the medium, was tested for amylase production, activity, and stability by* Bacillus tequilensis* RG-01 at their optimal temperature and incubation period.* Bacillus tequilensis *RG-01 showed 5200 U/mL enzyme productions, 100% activity, and 127% stability at pH 7.0 (Figure 4). Most of the* Bacillus* strains used commercially for the production of bacterial *α*-amylases have an optimum pH between 6.0 and 7.0 for growth and enzyme production [4, 18]. Strain RG-01 showed 86% activity and 91% stability at pH 9.0 (Figure 4). Haq et al. [21] reported pH 7.5–8.0 to be the best for the production of alpha amylase by* Bacillus subtilis*. Further increase and decrease in the medium pH reduced the enzyme production, activity, and stability (Figure 4). The pH change observed during the growth of the organism also affects product stability in the medium. Results show that enzyme production was generally active and stable from pH 5.0 to 10.0, which indicates excellent buffering property. The pH values also serve as a valuable indicator of the initiation and end of enzyme synthesis [22].

### 3.5. Effect of Different Incubation Periods

**Figure 5:**
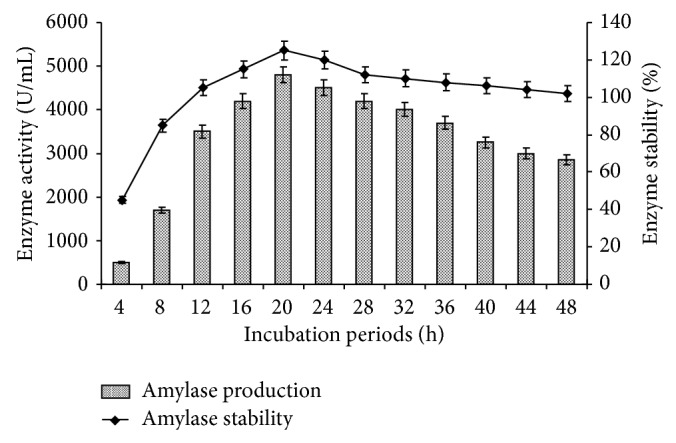
Effect of incubation periods on amylase production and stability. The flasks were inoculated with culture and were incubated at different incubation periods (4–48 h) at initial pH 7.0, 55°C. For enzyme activity, the reaction was assayed at respective incubation periods and for stability enzyme was preincubated for 4–48 h at 55°C for 1 h and assayed by standard assay method. Error bars presented mean values of ±standard deviation of triplicates of three independent experiments.

Just after optimization of temperature for amylase production, in the liquid medium, incubation period was optimized for enzyme production and stability. The results clearly indicated that* Bacillus tequilensis* RG-01 showed 4800 U/mL enzyme activity with 125% stability in 20 h of incubation (Figure 5). Further increase in the incubation period did not increase the enzyme production, but the stability of enzyme is 100% in 72 h (data not shown). In contrast to our results, Özdemir et al. [23] reported that *α*-amylase production by* B. subtilis* was maximum (2902 U/mg) in 72 h, after which a gradual decrease was observed. It may be due to denaturation or decomposition of *α*-amylase owing to interaction with other components in the medium, as it is reported elsewhere [24]. Incubation time depends on the characteristics of the culture, on growth rate, and enzyme production [25]. Moreover, the reaction for maximum enzyme production at 72 h could be due to the fact that the microorganism was in its exponential phase. At later stage, when nutrients are depleted, it reaches its stationary phase and can start to produce secondary metabolites, thus resulting in a lower yield of enzyme [26]. Thus, our strain producing *α*-amylase within 20 h of incubation is better than that reported by the other workers mentioned above.

### 3.6. Effect of Different Carbon Sources

**Figure 6:**
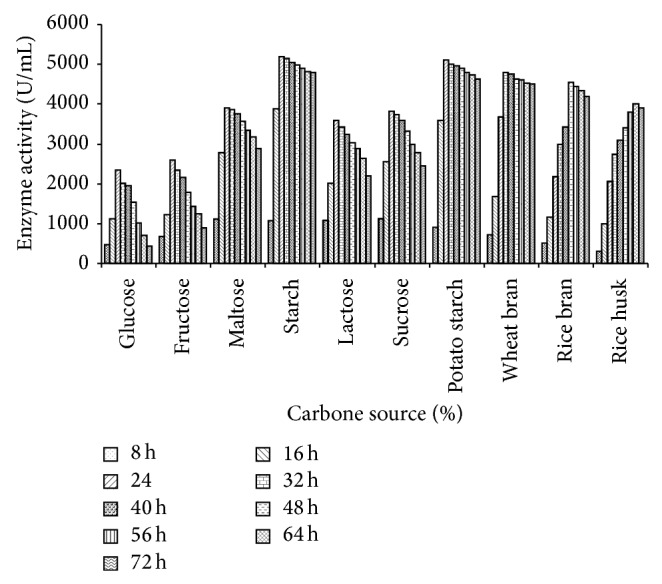
Effect of different carbon sources and incubation periods on amylase production. Test flasks contained different carbon sources in the medium at a level of 2% (w/v). The flasks were inoculated with culture and incubated at 55°C for 4–48 h at pH 7.0. Error bars presented are mean values of ±standard deviation of triplicates of three independent experiments.

Various carbon sources,* viz*, starch, wheat bran, rice bran, rice husk, potato starch, glucose, fructose, lactose, maltose, and sucrose at a concentration of 2.0% (w/v) were individually tested in the basal medium at their optimal temperature, incubation period, and pH to observe the effect on enzyme production by* Bacillus tequilensis *RG-01. Out of these carbon sources, starch (soluble) was found to be the best for amylase production (5200 U/mL) by the strain RG-01 followed by potato starch within 24 h (Figure 6). Similarly, Gangadharan et al. [27] reported that* Bacillus amyloliquefaciens* gave the highest enzyme yield (62470 U/g) with soluble starch, followed by maltose (58499 U/g).

*Bacillus tequilensis *RG-01 also showed maximum enzyme production (4800, 4550, and 4010 U/mL) in the presence of wheat bran, rice bran, and rice husk within 32, 48, and 64 h of incubation (Figure 6). Anto et al. [17] also reported that* B. cereus *using wheat bran showed highest production (122 ± 5 U/g).* Bacillus tequilensis *RG-01 showed considerable enzyme production with maltose, lactose, and sucrose within 24 h of incubation (Figure 6). Amylase production in the presence of maltose, lactose, and sucrose was also reported by some other workers [28].* Bacillus tequilensis* RG-01 showed minimum enzyme production in the presence of glucose and fructose (Figure 6). Some workers reported that supplementation of glucose caused a negative effect on amylase production [23, 29]. Glucose was found to repress the enzyme yield, which may be due to feedback inhibition caused by the presence of reducing sugars. Easily metabolizable carbohydrates may result in better growth of the bacteria along with reduction in the enzyme formation [30, 31].

**Figure 7:**
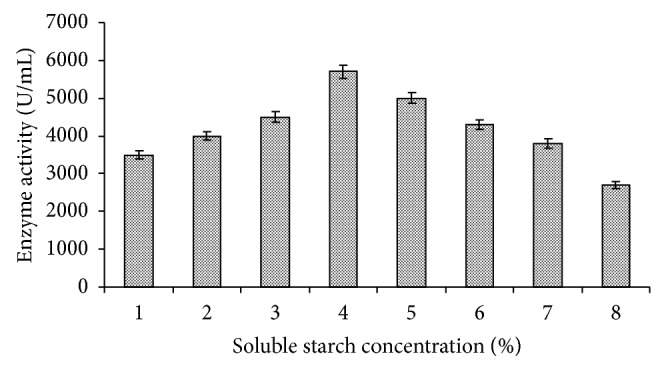
Effect of different concentration of soluble starch on amylase production. Test flasks contained different concentrations of starch (1.0–8.0, w/v) in the medium. The flasks were inoculated with culture and incubated at 55°C for 24 h at pH 7.0. Error bars presented are mean values of ±standard deviation of triplicates of three independent experiments.

In another set of the experiment, different concentrations of starch (1.0, 2.0, 3.0, 4.0, 5.0, 6.0, 7.0, and 8.0%, w/v) in the medium were tested for amylase production at the same growth conditions at which carbon sources were evaluated.* Bacillus tequilensis *RG-01 showed 5700 U/mL amylase production at 4% starch (soluble) concentration; above this concentration enzyme production was slightly decreased (Figure 7).

### 3.7. Effect of Different Nitrogen Sources

**Figure 8:**
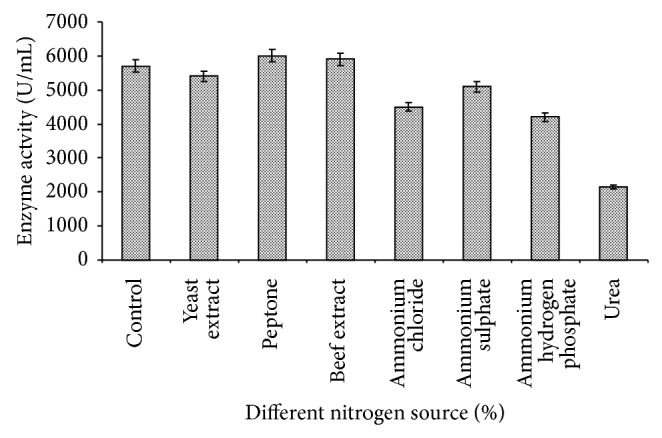
Effect of different nitrogen sources on amylase production. The control flask does not contain any nitrogen sources. Test flasks contained different nitrogen sources in the medium at a level of 0.5% (w/v). The flasks were inoculated with culture and incubated at 55°C for 24 h at pH 7.0 with 4.0% starch. Error bars presented are mean values of ±standard deviation of triplicates of three independent experiments.

Inorganic and organic nitrogen,* viz*, peptone, beef extract, yeast extract, urea, ammonium sulphate, ammonium chloride, and ammonium hydrogen phosphate, at the rate of 0.5% (w/v) were used in the basal medium for amylase production by* Bacillus tequilensis *RG-01 (Figure 8). The enzyme production by the isolate was almost similar in beef extract and peptone amended medium (6000 U/mL) followed by yeast extract and ammonium sulphate. Similarly, Thippeswamy et al. [32] reported that the addition of peptone increases amylase production by* Bacillus* sp.* Bacillus tequilensis* RG-01 also showed better amylase production in the presence of ammonium chloride and ammonium hydrogen phosphate. Narang and Satyanarayana [28] also reported that ammonium chloride and ammonium hydrogen phosphate favoured growth and enzyme secretion by bacterial strains. Other nitrogen source, like urea, showed inhibitory effect on amylase production of* Bacillus tequilensis* RG-01. Ramachandran et al. [26] have already reported that supplementation of urea at 1% concentration resulted in a decrease in amylase production.

**Figure 9:**
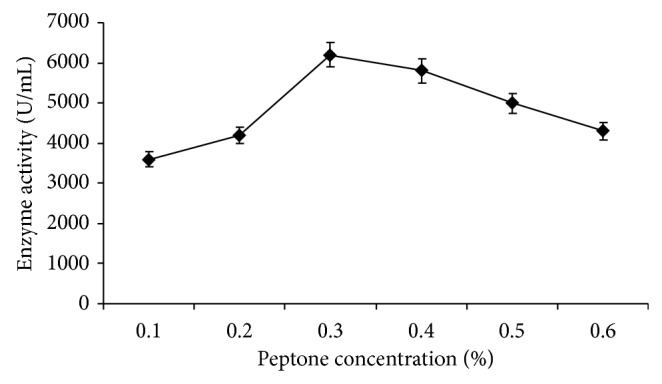
Effect of different concentration of peptone on amylase production. Test flasks contained different concentration of peptone (0.1–0.6%, w/v) in the medium. The flasks were inoculated with culture and incubated at 55°C for 24 h at pH 7.0. Error bars presented are mean values of ±standard deviation of triplicates of three independent experiments.

Different concentrations of peptone (0.1, 0.2, 0.3, 0.4, 0.5, and 0.6%, w/v) in the medium were also tested for amylase production at the same growth condition at which nitrogen sources were evaluated. Bacteria showed higher enzyme production (6200 U/mL) at 0.3% peptone concentration, further increase in concentration, reduced enzyme production (Figure 9).

### 3.8. Effect of Organic Solvents on Amylase Stability

In another approach, the effect of various organic solvents (30%, v/v) on amylase stability was also investigated for 7 days, and the results are depicted in Table 2. The amylase of* Bacillus tequilensis* RG-01 is extraordinarily stable in the presence of all organic solvents under study. It was observed that except for benzene, methanol, and ethanol, presence of other solvents enhanced the amylase activity. After incubation with n-dodecane, isooctane, n-decane, xylene, toluene, n-hexane, n-butanol, and cyclohexane, the amylase activity increased to 232, 160, 168.8, 120, 170.2, 133, 144, and 120%, respectively. The presence of benzene, methanol, and ethanol marginally reduced the amylase with residual activities of 72, 93.3, and 78.3%, respectively (Table 2). An organic solvent stable alkaline protease has been reported from* P. aeruginosa* PseA by Gupta and Khare [33]. After 10 days of incubation with organic solvents (25%, v/v), the residual protease activities were 112, 75, 98, 92, 97, 94, 75, 90, 96, 102, and 104% in the presence of ethanol, 1-butanol, benzene, toluene, xylene, cyclohexane, hexane, heptane, isooctane, n-decane, and n-dodecane, respectively. Abusham et al. [34] also reported that protease of* B. subtilis* strain showed enhanced activity in the presence of organic solvents (25%, v/v). It is therefore evident from our study that amylase of* Bacillus tequilensis* RG-01 is remarkably stable in the presence of broad range hydrophilic as well as hydrophobic organic solvents employed in this study.

### 3.9. Effect of Different Metal Salts

**Figure 10:**
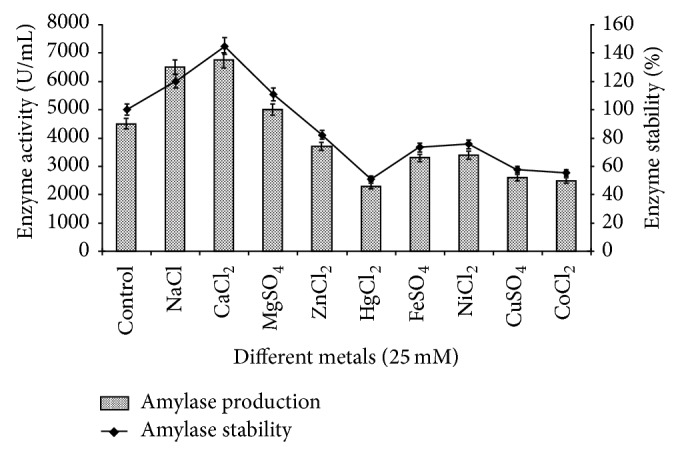
Effect of different metal ions on amylase production and stability. The control flask does not contain any metal ions. Test flasks contained different metal ions in the medium at a level of 25 mM. The flasks were inoculated with culture and were incubated at initial pH 7.0, 55°C for 24 h. For enzyme activity, the reaction was assayed and for stability enzyme was preincubated with different metal ions (25 mM) at 55°C for 1 h and assayed by standard assay method. Error bars presented mean values of ±standard deviation of triplicates of three independent experiments.

For the study of the effect of metal ions,* viz*, NaCl, CaCl_2_, MgSO_4_, HgCl_2_, FeSO_4_, NiCl_2_, CuSO_4_, CoCl_2_, and ZnCl_2_ at a concentration of 25 mM were individually tested in the basal medium at their optimal temperature, incubation period, and pH to observe the effect on enzyme production and stability by* Bacillus tequilensis *RG-01. Out of these metal ions, sodium and calcium ions were found to be the best for amylase production (6500 and 6750 U/mL) and stability (120 and 145%). While amylase production and stability of* Bacillus tequilensis *RG-01 were slightly reduced in the presence of Fe^2+^, Mg^2+^, Cu^2+^, Ni^2-^, Co^2+^, and Zn^2+^.* Bacillus tequilensis *RG-01 also tolerates 25 mM HgCl_2_, a novel finding of this strain (Figure 10). Amylolytic enzymes are metalloenzymes with up to six Ca^2+^ atoms at the active site whose catalytic activity can be affected by mono- and divalent metals [35]. Ca^2+^ enhanced the activity of *α*-amylase from* C. perfringens* [36],* B. subtilis* [37], and* Acremonium sporosulcatum* [38]. The inhibition of* B. subtilis* JS-2004 *α*-amylase by Co^2+^, Cu^2+^, and Ba^2+^ ions could be due to competition between the exogenous cations and the protein associated cations, resulting in decreased metalloenzymes activity [39]. Hernandez et al. [40] reported that amylase activity was strongly inhibited by Cu^2+^, Hg^2+^, and Zn^2+^.

**Figure 11:**
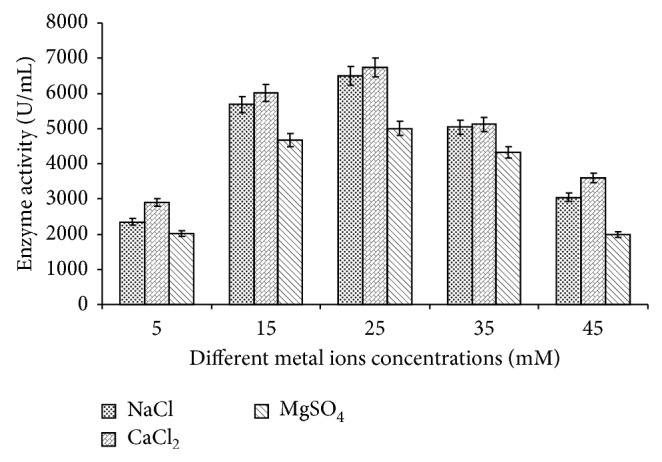
Effect of different concentration of metal ions (NaCl, CaCl_2_, and MgSO_4_) on amylase production. Test flasks contained different concentrations (5 mM to 45 mM) of metal ions (NaCl, CaCl_2_, and MgSO_4_) in the medium. The flasks were inoculated with culture and were incubated at initial pH 7.0, 55°C for 24 h. For enzyme activity, the reaction was assayed by standard assay method. Error bars presented mean values of ±standard deviation of triplicates of three independent experiments.

Different concentrations of NaCl, CaCl_2_, and MgSO_4_ (5, 10, 15, 25, 35, and 45 mM) in the medium were also tested for amylase production at the same growth condition at which metal ion sources were evaluated. Bacteria showed same pattern of amylase production with NaCl, CaCl_2_, and MgSO_4_ at 25 Mm concentration but maximum amylase production was reported from the CaCl_2_. By further increase and decrease in the concentration of all three metal ions, enzyme production was reduced (Figure 11).

### 3.10. Effect of Surfactants on Amylase Activity

**Figure 12:**
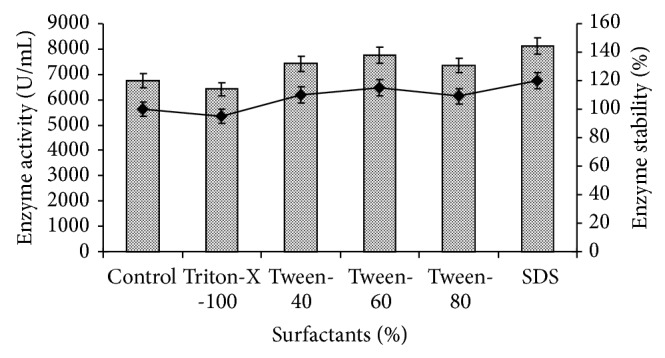
Effect of different surfactants on amylase production and stability. The control flask does not contain any surfactant. Test flasks contained different surfactant in the medium at a level of 1.0% (w/v). The flasks were inoculated with culture and were incubated at initial pH 7.0, 55°C for 24 h. For enzyme activity, the reaction was assayed and for stability enzyme was preincubated with different surfactants (1.0%) at 55°C for 1 h and assayed by standard assay method. Error bars presented mean values of ±standard deviation of triplicates of three independent experiments.

None of the surfactants tested had a pronounced inhibitory effect on enzyme activities. Only Triton-X-100 showed a moderate inhibitory effect (5%) on amylase activity of* Bacillus tequilensis *RG-01 (Figure 12). On the other hand, the addition of SDS, Tween-40, Tween-60, and Tween-80 (1%) was found to stimulate activity by 20, 10, 15, and 9%, respectively. The stimulatory effect of surfactants on amylase activity is controversial. Oberoi et al. [41] also reported that the activity of amylase of* Bacillus *sp. was stimulated in the presence of Tween-80 (1%). Arnesen et al. [42] reported that amylase activity of* Thermomyces lanuginosus* was slightly inhibited by Triton X-100. A stimulatory effect of SDS (6%) on amylase activity and stability in* Bacillus* sp. strain TS-23 was documented by Lo et al. [43]. From the previous reports and the present study, it was evident that the same surfactant could have different effects on the activity of the same enzyme in the same microorganism or on the activity of different enzymes in the same microorganism.

### 3.11. SDS-PAGE Analysis of Amylase Enzyme

**Figure 13:**
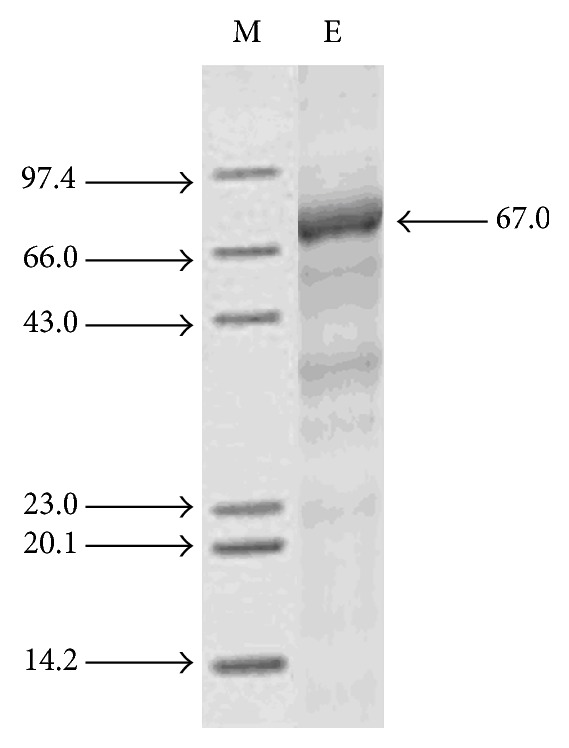
Photographic representation of the SDS-PAGE of amylase of* Bacillus tequilensis*. Lane 1: marker proteins; lane 2: enzyme supernatant (amylase). Molecular weights were presented in the form of kDa.

The purity of the enzyme was confirmed by the presence of a single band on SDS-PAGE and its molecular weight was approximately 67 kDa (Figure 13), which was similar to* Bacillus subtilis* amylase (67 kDa) [44] but different from* Bacillus *sp. YX-1 amylase (56 kDa) [45].

## 4. Conclusion

A thermotolerant solvent stable amylase is produced by a novel isolate* Bacillus tequilensis* RG-01. The organism appears to have greater potential for enhanced enzyme production through optimization of nutritional and physical parameters. Tolerance against temperature, pH, organic solvents, metal ions, and surfactants facilitates its use for various processes under stressed conditions. Owing to its (*Bacillus tequilensis *RG-01) thermotolerant nature, its amylase may have potential uses in industries such as detergent, food, pharmaceutical, leather, and agriculture, as well as molecular biology techniques.

## Figures and Tables

**Table 1:** Morphological, physiological, and biochemical characteristics of the selected RG-01 isolate.

Characteristics	Results
Morphological tests	
Gram's reaction	+
Shape	Cylindrical
Motility	+

Physiological tests	
Growth on NaCl (%) 2.0–8.0	+
Growth at pH 7.0–12.0	+
Growth at temp. 25–95°C	+
Growth under anaerobic condition	+

Biochemical tests	
Growth on MacConkey agar	−
Indole test	−
Methyl red test	−
Voges-Proskauer test	+
Citrate utilization	−
H_2_S production	−
Gelatin hydrolysis	+
Esculin hydrolysis	+
Starch hydrolysis	+
Urea hydrolysis	−
Nitrate reduction	−
Ornithine decarboxylase	−
Lysine decarboxylase	−
Catalase test	+
Oxidase test	+
Arginine decarboxylase	−
Tween-20 hydrolysis	+
Tween-40 hydrolysis	+
Gas production from glucose	−

Acid production from	
Dextrose	+
Lactose	−

(+): positive; (−): negative.

**Table 2:** Stability of crude amylase in presence of various organic solvents.

Organic solvents (30%)	log⁡*P*	Residual activity (%)							
		1 h	24 h	48 h	72 h	96 h	120 h	144 h	168 h
Methanol	−0.76	101.9	130.5	133.2	125.3	120.6	113.4	106.3	93.3
Isopropanol	−0.28	90.2	100.3	96.3	90.4	89.5	86.4	80.3	75.4
Ethanol	−0.24	97	105.6	103.5	100.6	96.5	90.4	81.2	78.3
Benzene	2.13	99	110	95	90	86	81	78	72
Cyclohexane	3.3	87	105	120	102	99	91	80	75
Acetone	−0.23	101.5	105.4	103.9	100.8	95	90	85	80
Butanol	−0.80	112.7	144.3	128.2	115.4	107.6	100.3	100	95
Toluene	2.5	115.9	170.2	140.1	127.1	120.8	112.9	105.5	100.3
Isooctane	2.9	120	160.1	125.5	120.5	113.4	110.3	105.7	99.2
Xylene	3.1	89	107	120	110	106	100	94	91
Hexane	3.6	114	133	124	118	113	104	100	100
n-Decane	5.6	125.3	151.3	168.8	148.3	126.2	101.7	100	99.0
n-Dodecane	6.0	131.8	160.2	232.3	158.1	149.9	120.2	108.0	100.0

Enzyme was preincubated with different organic solvents at a concentration of 30% (v/v) at 55°C for different time periods and assayed as standard assay method. The enzyme activity without incubation with organic solvent was taken as 100%. Mean standard deviation for all values is <±5.0%.

## References

[1] van der Maarel M. J., van der Veen B., Uitdehaag J. C., Leemhuis H., Dijkhuizen L. (2002). Properties and applications of starch-converting enzymes of the *α*-amylase family. *Journal of Biotechnology*.

[2] Burhan A., Nisa U., Gökhan C., Ömer C., Ashabil A., Osman G. (2003). Enzymatic properties of a novel thermostable, thermophilic, alkaline and chelator resistant amylase from an alkaliphilic *Bacillus* sp. isolate ANT-6. *Process Biochemistry*.

[3] Aiyer P. V. (2005). Amylases and their applications. *African Journal of Biotechnology*.

[4] Vidyalakshmi R., Paranthaman R., Indhumathi J. (2009). Amylase production on submerged fermentation by Bacillus sp.. *World Journal of Chemistry*.

[5] Maton A., Jean H., Charles W., Susan J., Maryanna Q., David L., Jill D. (1993). *Human Biology and Health*.

[6] Ajayi A. O., Fagade O. E. (2003). Utilization of corn starch as substrate for *β*-amylase by *Bacillus* sp.. *African Journal of Biomedical Research*.

[7] Kathiresan K., Manivannan S. (2006). *α*-Amylase production by *Penicillium fellutanum* isolated from mangrove rhizosphere soil. *African Journal of Biotechnology*.

[8] El Enshasy H. A. (2007). Bioprocess development for the production of *α*-amylase by *Bacillus amyloliquefaciens* in batch and fed-batch cultures. *Research Journal of Microbiology*.

[9] Konsoula Z., Liakopoulou-Kyriakides M. (2006). Thermostable *α*-amylase production by *Bacillus subtilis* entrapped in calcium alginate gel capsules. *Enzyme and Microbial Technology*.

[10] Yun J., Kang S., Park S., Yoon H., Kim M.-J., Heu S., Ryu S. (2004). Characterization of a novel amylolytic enzyme encoded by a gene from a soil-derived metagenomic library. *Applied and Environmental Microbiology*.

[11] Chengyi W. H., Ming M., Jiang R. (1999). Studies on the properties of alpha-amylase produced by *Bacillus pumilus* 289 (PBX96). *Acta Microbiologica Sinica*.

[12] Creig R. N., Holt G. J. (1984). *Bergey's Manual of Systematic Bacteriology*.

[13] Nelson N. (1944). A photometric adaptation of the Somogyi method for the determination of glucose. *The Journal of Biological Chemistry*.

[14] Somogyi M. (1952). Notes on sugar determination. *The Journal of Biological Chemistry*.

[15] Laemmli U. K. (1970). Cleavage of structural proteins during the assembly of the head of *bacteriophage* T4. *Nature*.

[16] Gupta R., Beg Q., Lorenz P. (2002). Bacterial alkaline proteases: molecular approaches and industrial applications. *Applied Microbiology and Biotechnology*.

[17] Anto H., Trivedi U., Patel K. (2006). Alpha amylase production by *Bacillus cereus* MTCC 1305 using solid-state fermentation. *Food Technology and Biotechnology*.

[18] Soni S. K., Kaur A., Gupta J. K. (2003). A solid state fermentation based bacterial *α*-amylase and fungal glucoamylase system and its suitability for the hydrolysis of wheat starch. *Process Biochemistry*.

[19] Gupta R., Gigras P., Mohapatra H., Goswami V. K., Chauhan B. (2003). Microbial *α*-amylases: a biotechnological perspective. *Process Biochemistry*.

[20] Pedersen H., Nielsen J. (2000). The influence of nitrogen sources on the *α*-amylase productivity of *Aspergillus oryzae* in continuous cultures. *Applied Microbiology and Biotechnology*.

[21] Haq I. U., Ashraf H., Iqbal J., Qadeer M. A. (2002). Biosynthesis of *α*-amylase by chemically treated mutant of *Bacillus subtilis*. *Online Journal of Biological Sciences*.

[22] Friedrich J., Cimerman A., Steiner W. (1989). Submerged production of pectolytic enzymes by *Aspergillus niger*: effect of different aeration/agitation regimes. *Applied Microbiology and Biotechnology*.

[23] Özdemir S., Güven K., Baysal Z., Uyar F. (2009). Screening of various organic substrates and the development of a suitable low-cost eermentation medium for the production of *α*-amylase by *Bacillus subtilis*. *Food Technology and Biotechnology*.

[24] Ramesh M. V., Lonsane B. K. (1987). A novel bacterial thermostable alpha-amylase system produced under solid state fermentation. *Biotechnology Letters*.

[25] Baysal Z., Uyar F., Aytekin Ç. (2003). Solid state fermentation for production of *α*-amylase by a thermotolerant *Bacillus subtilis* from hot-spring water. *Process Biochemistry*.

[26] Ramachandran S., Patel A. K., Nampoothiri K. M., Francis F., Nagy V., Szakacs G., Pandey A. (2004). Coconut oil cake—a potential raw material for the production of *α*-amylase. *Bioresource Technology*.

[27] Gangadharan D., Sivaramakrishnan S., Nampoothiri K. M., Pandey A. (2006). Solid culturing of *Bacillus amyloliquefaciens* for alpha amylase production. *Food Technology and Biotechnology*.

[28] Narang S., Satyanarayana T. (2001). Thermostable *α*-amylase production by an extreme thermophile *Bacillus thermooleovorans*. *Letters in Applied Microbiology*.

[29] Mulimani V. H., Ramalingam G. N. P. (2000). *α*-Amylase production by solid state fermentation: a new practical approach to biotechnology courses. *Biochemical Education*.

[30] Yoon M. Y., Yoo Y. J., Cadman T. W. (1989). Phosphate effects in the fermentation of *α*-amylase by *Bacillus amyloliquefaciens*. *Biotechnology Letters*.

[31] Rama R., Srivastav S. K. (1995). Effect of various carbon substrates on *α*-amylase production from *Bacillus* species. *Journal of Microbiology and Biotechnology*.

[32] Thippeswamy S., Girigowda K., Mulimani V. H. (2006). Isolation and identification of *α*-amylase producing *Bacillus* sp. from dhal industry waste. *Indian Journal of Biochemistry and Biophysics*.

[33] Gupta A., Khare S. K. (2007). Enhanced production and characterization of a solvent stable protease from solvent tolerant *Pseudomonas aeruginosa* PseA. *Enzyme and Microbial Technology*.

[34] Abusham R. A., Rahman R. N. Z. R. A., Salleh A., Basri M. (2009). Optimization of physical factors affecting the production of thermo-stable organic solvent-tolerant protease from a newly isolated halo tolerant *Bacillus subtilis* strain Rand. *Microbial Cell Factories*.

[35] Normurodova K. T., Nurmatov S. K., Alimova B. K., Pulatova O. M., Akhmedova Z. R., Makhsumkhanov A. A. (2007). Isolation and characteristics of highly active *α*-amylase from *Bacillus subtilis*-150. *Chemistry of Natural Compounds*.

[36] Shih N. J., Labbe R. G. (1995). Purification and characterization of an extracellular *α*-amylase from *Clostridium perfringens* type A. *Applied and Environmental Microbiology*.

[37] Asgher M., Asad M. J., Rahman S. U., Legge R. L. (2007). A thermostable *α*-amylase from a moderately thermophilic *Bacillus subtilis* strain for starch processing. *Journal of Food Engineering*.

[38] Valaparla V. K. (2010). Purification and properties of a thermostable *α*-amylase by *Acremonium Sporosulcatum*. *International Journal of Biotechnology and Biochemistry*.

[39] Lévêque E., Janeček Š., Haye B., Belarbi A. (2000). Thermophilic archaeal amylolytic enzymes. *Enzyme and Microbial Technology*.

[40] Hernandez M. S., Rodriguez M. R., Guerra N. P., Roses R. P. (2006). Amylase production by *Aspergillus niger* in submerged cultivation on two wastes from food industries. *Journal of Food Engineering*.

[41] Oberoi R., Beg Q. K., Puri S., Saxena R. K., Gupta R. (2001). Characterization and wash performance analysis of an SDS-stable alkaline protease from a *Bacillus* sp. *World Journal of Microbiology and Biotechnology*.

[42] Arnesen S., Eriksen S. H., Olsen J. Ø., Jensen B. (1998). Increased production of *α*-amylase from Thermomyces lanuginosus by the addition of Tween 80. *Enzyme and Microbial Technology*.

[43] Lo H.-F., Lin L.-L., Chen H.-L., Hsu W.-H., Chang C.-T. (2001). Enzymic properties of a SDS-resistant *Bacillus* sp. TS-23 *α*-amylase produced by recombinant Escherichia coli. *Process Biochemistry*.

[44] Yandri Suhartati T., Hadi S. (2010). Purification and characterization of extracellular α-amilase enzyme from locale bacteria isolate *Bacillus subtilis* ITBCCB148. *European Journal of Scientific Research*.

[45] Liu X. D., Xu Y. (2008). A novel raw starch digesting *α*-amylase from a newly isolated *Bacillus sp*. YX-1: purification and characterization. *Bioresource Technology*.

